# Instance dataset for a multiprocessor scheduling problem with multiple time windows and time lags: Similar instances with large differences in difficulty

**DOI:** 10.1016/j.dib.2022.108687

**Published:** 2022-10-21

**Authors:** Emil Karlsson, Elina Rönnberg

**Affiliations:** a Department of Mathematics, Linköping University, SE-581 83 Linköping, Sweden; b Saab AB, SE-581 88 Linköping, Sweden

**Keywords:** Multiprocessor scheduling, Constraint programming, Multiple time windows, Time lags, Exact time lags, Instances, Instance dataset, Avionics scheduling

## Abstract

The dataset presented in this paper introduces 384 new instances for the feasibility version of a multiprocessor scheduling problem with multiple time windows, positive time lags and exact time lags. The instances are constructed from subproblems in a logic-based Benders decomposition scheme introduced in “Logic-based Benders decomposition with a partial assignment acceleration technique for an avionics scheduling problem” (Karlsson, E., Rönnberg, E., Computers & Operations Research, 2022) [1]. A key aspect of the dataset is that even if two instances are highly similar, the computational performance of solving them with an IBM ILOG CP Optimizer model can be vastly different. There exists for example 44 pairs of instances with the same number of tasks and exact time lags, and the number of positive time lags differs with at most two, where one instance can be solved within 5 minutes and the other instance cannot be solved within 24 hours. Such differences make the instance dataset useful for investigating differences in computational performance of constraint programming solvers. The dataset can also be used to benchmark methods for multiprocessor scheduling. The dataset has been released under the Creative Commons Attribution 4.0 International license and can be used as it is or be adapted.

**Specifications Table**

**Table:** 

Subject	Control and Optimization
Specific subject area	Multiprocessor scheduling instances with multiple time windows, positive time lags and exact time lags
Type of data	Instance dataset
How the data were acquired	The instance dataset has been constructed from subproblems solved during the computational tests of the logic-based Benders decomposition method introduced in [1] for solving the avionics scheduling instances introduced in [2] for the problem studied in [1][2][3].
Data format	Raw Analyzed
Description of data collection	Instances for the described multiprocessor scheduling problem were derived from the experiments in [1] by transforming subproblems instances that from [1] were known to have similar properties but resulting in vastly different computational times (IBM ILOG CP Optimizer). The transformed instances were then analysed to ensure that the computational properties of the original subproblems were preserved.
Data source location	The dataset can be accessed via two locations: - Via a persistent institutional repository at https://doi.org/10.48360/etww-2281 - Via a version-controlled repository at https://gitlab.liu.se/eliro15/multiprocessor_scheduling_inst
Data accessibility	The dataset can be accessed via two locations: - Via a persistent institutional repository at https://doi.org/10.48360/etww-2281 - Via a version-controlled repository at https://gitlab.liu.se/eliro15/multiprocessor_scheduling_inst
Related research article	E. Karlsson, E. Rönnberg, Logic-based Benders decomposition with a partial assignment acceleration technique for an avionics scheduling problem, Computers & Operations Research. In Press, 2022, https://doi.org/10.1016/j.cor.2022.105916.

## Value of the Data

• This instance dataset contains 384 multiprocessor scheduling instances divided into 23 groups that illustrate that minor differences in the instances make major differences in computational performance for IBM ILOG CP Optimizer.
• Someone who want to study the robustness of constraint programming solvers or needs a challenging instance dataset to benchmark their methods on can benefit from this instance dataset.
• Further insights can be obtained by investigating and understanding the differences in the performance of the used CP solver.
• Insights on how to solve instances in this dataset could contribute to the development of optimisation methods for multiprocessor and avionics scheduling problems.

## 1 Objective

The main reason for generating this dataset was the observation that even if two instances of the multiprocessor scheduling problem with multiple time windows and time lags were highly similar, the computational performance of solving them with IBM ILOG CP Optimizer could be vastly different. This observation was made during the development and the computational testing of “Logic-based Benders decomposition with a partial assignment acceleration technique for an avionics scheduling problem” (Karlsson, E., Rönnberg, E., Computers & Operations Research, 2022) [1]. In the computational study of [1], this computational aspect was noted but not further addressed.

## 2 Data Description

This paper introduces a dataset that contains 384 instances for the feasibility version of a multiprocessor scheduling problem with multiple time windows, positive time lags and exact time lags. Particular for the dataset is that even though two instances are constructed almost identically, the computational difficulty of solving the instances can be vastly different. There exists for example 44 instance pairs that has the same number of tasks and exact time lags where the number of positive time lags differs with two, where one of the instances can be solved within 5 minutes and the other one cannot be solved in 24 hours with IBM ILOG CP Optimizer. The finding of such instance pairs is the main reason for publishing the instance dataset.

### 2.1 Problem formulation

This section includes a formal description of the feasibility version of the multiprocessor scheduling problem with multiple time windows, positive time lags and exact time lags.

This problem is a simplification of the relaxation subproblem used in the Logic-Based Benders Decomposition (LBBD) scheme of [1] used to solve an avionics scheduling problem studied in [1,2,3]. The simplification has been constructed so that the main characteristics and the computational behaviour of the relaxation subproblem is kept, but some of the technical details related to avionics scheduling are omitted. The details of the simplification are described in Section 2.1.

Before defining the requirements of a feasible solution, we present the notation used to describe a problem instance. First off, each instance contains a list of processors H. For each processor h∈H, there is a list of tasks $I_{h}$. For each task $i\in I_{h}$, on processor h∈H, a processing time $p_{i}\in Z_{0}^{+}$ and a list of time windows $Q_{i}$ are provided. For each time window $q\in Q_{i}$ of task i∈I on processor h∈H, a release time $r_{iq}\in Z_{0}^{+}$ and a deadline $d_{iq}\in Z_{0}^{+}$ are given. After the processors, there is a list of task pair indices $T^{p}\subseteq \left[\left(i,\;i^{\prime }\right)\in \;\cup_{h\in H}I_{h}\times \cup_{h\in H}I_{h}\right]$ referred to as positive time lags. For each positive time lag $\left(i,\;i^{\prime }\right)\in \;T^{p}$, there is a minimum length $l_{ii{}^{\prime }}\in Z_{0}^{+}$. Finally, there is a list of task pair indices $T^{e}\subseteq \left[\left(i,\;i^{\prime }\right)\in \;\cup_{h\in H}I_{h}\times \cup_{h\in H}I_{h}\right]$ referred to as exact time lags. For each exact time lag $\left(i,\;i^{\prime }\right)\in \;T^{e}$, there is an exact length $l_{ii{}^{\prime }}\in Z_{0}^{+}$. For each positive time lag and exact time lag $\left(i,\;i^{\prime }\right)\in \;T^{p}\cup T^{e}$, we let task index i be called the start task and task index $i{}^{\prime }$ be called the end task.

A feasible solution to the multiprocessor scheduling problem with multiple time windows, positive time lags and exact time lags is a schedule that satisfies certain constraints. A schedule for a problem instance is a list $S=[s_{i}\in Z_{0}^{+}:\;i\in I_{h},\;h\in H]$ of integer start times that describes the processing start of each task $i\in I_{h}$ on processor h∈H. A schedule is feasible for a problem instance if the following requirements are fulfilled.

**Req. 1.** Each task $i\in I_{h}$ on processor h∈H is non-preemptively scheduled within one of its time windows $q\in Q_{i}$ for the duration of its processing time $p_{i}$. A task $i\in I_{h}$ on processor h∈H is scheduled within its time window $q\in Q_{i}$ if $s_{i}\geq r_{iq}$ and $s_{i}+\;p_{i}\leq d_{iq}$.
**Req. 2.** On each processor h∈H, at most one task is processed at the same time. Hence, for each time step $t\in Z_{0}^{+}$, the inequality $|\left\{i_{h}:\;s_{i}\leq t<s_{i}+p_{i}\right\}|\leq 1$ must hold. Note that for each time step, it is allowed that a task end its processing and another task starts its processing.
**Req. 3.** For each positive time lag $\left(i,\;i^{\prime }\right)\in T^{p}$, task $i{}^{\prime }$ must start at least $l_{ii{}^{\prime }}$ time steps after the start of task i. Hence, $s_{i}\leq s_{i^{\prime }}+l_{ii{}^{\prime }}$ must hold for each positive time lag $\left(i,\;i^{\prime }\right)\in \;T^{p}$.
**Req. 4.** For each exact time lag $\left(i,\;i^{\prime }\right)\in T^{e}$, task $i{}^{\prime }$ must start exactly $l_{ii{}^{\prime }}$ time steps after the start of task i. Hence, $s_{i}=s_{i{}^{\prime }}+l_{ii{}^{\prime }}$ must hold for each exact time lag $\left(i,\;i^{\prime }\right)\in \;T^{e}$.

### 2.2 Constraint programming model

To analyse the computational difficulty of the instances, we formulated a Constraint Programming (CP) model for the multiprocessor problem defined in Section 1.1 and solved it with IBM ILOG CP Optimizer. The CP model used to test if a problem instance is feasible or infeasible is

min0

(1) $$s.t.\;\text{ForbidStart}\left(y_{i},\;Q_{i}\left(t\right)\right),\;i\in I_{h},\;h\in H,$$

(2) $$\text{StartBeforeStart}(y_{i},\;y_{i^{\prime }},\;l_{ii^{\prime }}),\;\left(i,\;i^{\prime }\right)\in T^{p},\;$$

(3) $$\text{StartAtStart}(y_{i},\;y_{i^{\prime }},\;l_{ii^{\prime }}),\;\left(i,\;i^{\prime }\right)\in T^{e},\;$$

(4) $$\text{Disjunctive}\left((y_{i}\;|\;i\in I_{h}\right),\;\left(e_{i}\;|\;i\in I_{h}\right)),\;h\in H,\;$$

where for each task i∈I, the interval variable $y_{i}$ represent its start time and $Q_{i}\left(t\right)$ is a step function that, for each time point t, takes the value 1 if $t\in \;\cup_{q\in Q_{i}}\left[r_{iq},d_{iq}\right]$ and 0 otherwise. Constraints (1), (2), (3), (4) ensure that Requirements 1–4 of a feasible schedule, respectively, are obeyed.

An instance is called easy or medium if the solution time was less than 5 minutes or between 5 minutes and 24 hours, respectively. If an instance was not solved within 24 hours, it is called hard. There are 176 hard problem instances in the instance dataset and for these, it has currently *not* been determined if the problem instance is feasible or infeasible. Hence, the instance dataset can be used to benchmark methods for solving multiprocessor scheduling problems.

**Fig. 1:**
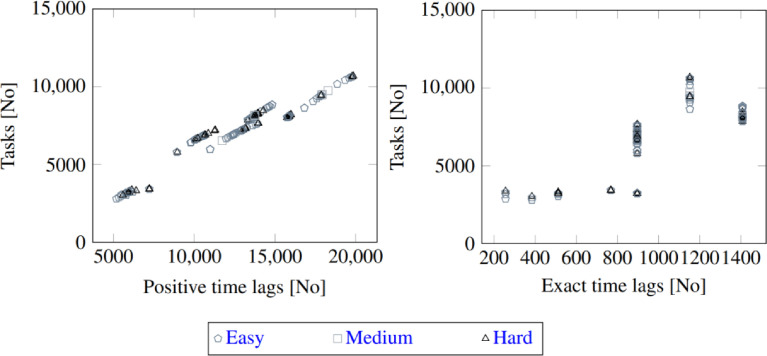
Relationship between the number of tasks, positive time lags and exact time lags in the instance dataset

Fig. 1 illustrates the number of tasks, positive time lags, and exact time lags for the instance dataset divided into easy, medium, and hard instances.

### 2.3 Instance groups

Each instance in the dataset is constructed from a subproblem encountered when applying the LBBD scheme in [1] to a public avionics scheduling instance introduced in [2]. To construct an instance in the dataset, a subset of the master problem variables in the LBBD scheme are fixed to their values in a master problem solution. The impact of these master problem decisions is propagated to the remaining variables that represent start times for tasks. After this, a separate subproblem is formed for each independent set of processors. We will be referring to a set of independent set of processors as a processor set.

In total, there are 23 combinations of master problem solutions and processor sets used to construct the instances in the dataset. We will be referring to the set of instances constructed from the same master problem solution and processor set as an instance group. In Table 1, the number of easy, medium and hard instances of each instance group in the dataset are listed. The table also details the original avionics scheduling instance, the processor set ID and LBBD iteration that each instance group was created from.

**Table 1:** The number of Easy (E), Medium (M) and Hard (H) instances in each instance group as well as the LBBD iteration, the processor set ID, and the original avionics scheduling instance that each instance group was constructed from

Group ID	Org. instance	LBBD iteration	Processor set ID	No. instances
1	InstanceA7	1	0	3 (2E, 0M, 1H)
2	InstanceB26	1	1	3 (2E, 0M, 1H)
3	InstanceC9	2	2	3 (2E, 0M, 1H)
4	InstanceD12	3	0	25 (15E, 3M, 7H)
5	InstanceD14	3	5	9 (2E, 0M, 7H)
6	InstanceD14	6	0	7 (2E, 0M, 5H)
7	InstanceD14	14	0	7 (3E, 0M, 4H)
8	InstanceD14	17	0	10 (3E, 0M, 7H)
9	InstanceD14	28	0	13 (8E, 0M, 5H)
10	InstanceD14	30	2	18 (10E, 0M, 8H)
11	InstanceD14	31	2	22 (10E, 0M, 12H)
12	InstanceD14	32	2	19 (9E, 0M, 10H)
13	InstanceD14	33	0	25 (16E, 0M, 9H)
14	InstanceD14	35	0	17 (9E, 0M, 8H)
15	InstanceD14	36	0	16 (8E, 0M, 8H)
16	InstanceD14	37	0	16 (15E, 0M, 1H)
17	InstanceD14	38	0	17 (5E, 0M, 12H)
18	InstanceD16	1	0	7 (5E, 0M, 2H)
19	InstanceD16	4	0	61 (26E, 6M, 29H)
20	InstanceD18	1	6	4 (3E, 0M, 1H)
21	InstanceD27	4	1	46 (31E, 1M, 14H)
22	InstanceD28	12	0	34 (20E, 0M, 14H)
23	InstanceD28	15	0	2 (1E, 0M, 1H)

In each instance group, there is at least one easy instance and at least one hard instance.

To simplify the analysis of instances within the same instance group, there is – for each instance on the website of the dataset – a text file that contains the subset of master problem variables that was fixed before constructing the subproblem. To give some idea about the spread of the instances belonging to the same group, the range of the number of tasks, positive time lags and exact time lags and the number of processors in each instance group are illustrated in Table 2. The instance group in the dataset with the largest number of instances is number 19 that has 61 instances where 26 are easy, 6 are medium, and 29 are hard.

**Table 2:** Range of the number of tasks, positive time lags and exact time lags and the number of processors for the instance groups in the dataset

Group ID	No. tasks	No. pos. time lags	No. exact time lags	No. processors
1	2794–3028	5181–5583	384–384	2
2	3059–3223	5485–5892	512–512	2
3	3034–3320	5791–6402	512–512	2
4	8637–10,691	16,821–19,855	1152–1152	2
5	3421–3433	7214–7230	768–768	2
6	8036–8042	15,759–15,769	1408–1408	2
7	8036–8040	15,758–15,766	1408–1408	2
8	8036–8046	15,759–15,828	1408–1408	2
9	8036–8064	15,758–15,804	1408–1408	2
10	3214–3242	5932–5971	896–896	3
11	3214–3238	5932–5968	896–896	3
12	3214–3234	5932–5962	896–896	3
13	8036–8132	15,758–15,904	1408–1408	2
14	8036–8206	15,758–15,988	1408–1408	2
15	8036–8206	15,758–15,988	1408–1408	2
16	8036–8206	15,759–15,988	1408–1408	2
17	8036–8206	15,758–15,985	1408–1408	2
18	7863–7865	13,323–13,329	1408–1408	2
19	7863–8847	13,323–15,832	1408–1408	2
20	2869–3373	5330–6133	256–256	2
21	5970–7658	10,983–13,972	896–896	2
22	5793–7171	8927–11,527	896–896	2
23	6837–7217	10,807–11,301	896–896	2

### 2.4 Easy-hard similar pairs

**Fig. 2:**
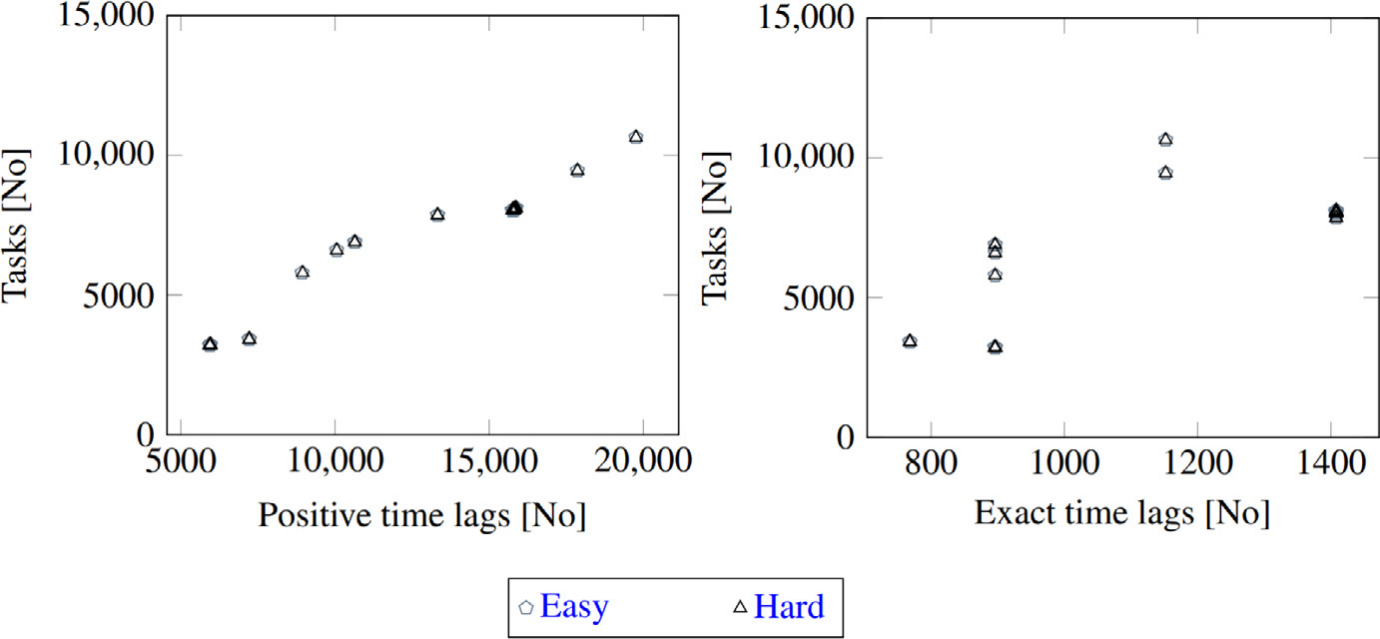
Relationship between the number of tasks, positive time lags and exact time lags in instances in the easy-hard similar pairs

A key aspect of the dataset is that even if the subset of variables used to construct an instance is similar, the computational difficulty of the instances can be vastly different. There exists for example 44 pairs of instances in the dataset where the instances in the pair belongs to the same instance group but the subset of variables that are fixed differs only in one variable, where one instance can be solved within 5 minutes and the other instance cannot be solved within 24 hours with IBM ILOG CP Optimizer. The set of such instance pairs are referred to as easy-hard similar pairs. All easy-hard similar pairs have the same number of tasks and exact time lags, and the number of positive time lags differ with at most two. Fig. 2 illustrates the number of tasks, positive time lags, and exact time lags for all instances in the easy-hard similar pairs. A comprehensive list of easy-hard similar pairs can be found at the website of the instance dataset.

### 2.5 Data format

Each problem instance is provided in a separate file. Each file is named according to the structure ‘g_iid_l_oid_c_h.zip’, where ‘g’, ‘iid’, ‘l’, ‘oid’ and ‘c’, denote the instance group id, instance id, LBBD iteration number, the original avionics scheduling instance in https://gitlab.liu.se/eliro15/avionics_inst, and processor set ID of an instance, respectively, and ‘h’ represent if the instance is easy, medium, or hard. For example, problem instance ‘1_25_12_InstanceD28_cmid0_hard.zip’ is a hard instance generated from processor set ID 0 of the 12^th^ LBBD iteration of InstanceA7 that belongs to instance group number 1 and has instance id 25. In addition to each instance file, there is also a support file ‘g_iid_l_oid_c_h.txt’ that contains the subset of master problem variables that were used to construct the instance. The instance dataset and code for performing the computational tests can be found at the version-controlled repository https://gitlab.liu.se/eliro15/multiprocessor_scheduling_inst or via an institutional repository at https://doi.org/10.48360/etww-2281.

### 2.5 Instance dataset license

The instance dataset is released under the Creative Commons Attribution 4.0 International license.

## 3 Experimental Design, Materials and Methods

The dataset is constructed during the computational testing of the LBBD scheme introduced in [1]. Each instance in the dataset is constructed by performing the following four steps.

(i) For a subset of the master problem variables, the value of the variable is fixed to that of the master problem solution from an LBBD scheme.
(ii) The impact of these master problem decisions is propagated to the remaining variables that represent start times for tasks.
(iii) A separate subproblem is formed for each independent set of processors.
(iv) Each separate subproblem is transformed into an instance for the feasibility version of a multiprocessor scheduling problem with multiple time windows, positive time lags, and exact time lags using the method in Section 2.1.

The first three steps are part of the LBBD scheme for the avionics scheduling problem in [1] and for a detailed description, we refer to the paper. The fourth step is used to remove some technical details in the subproblem to create instance for a more generic problem structure. This was only done to prepare the instance dataset.

To construct our instance set, we selected subproblems candidates from the computational tests of our LBBD scheme. In more detail, we identified all master problem solutions and processor sets that resulted in at least one subproblem that timed out (runned more than 5 minutes without any conclusion of feasibility or infeasibility) during the LBBD evaluation and selected all subproblems that were constructed from these combinations of master problem solutions and processors sets. This resulted in a total of 26 instance group candidates with a total of 652 subproblem candidates. For each subproblem in a candidate instance group, we performed Steps (i)–(iv) above and solved the resulting instance using the CP model in Section 1.2. Finally, we kept all instances from the candidate instance groups that contains at least one easy instance and one hard instance. This resulted in a total of 23 instance groups with a total of 384 instances.

The CP-model evaluations were performed using Python 3.8 and IBM ILOG CP Optimizer 12.20. The computational evaluation performed when preparing this instance dataset was carried out on a computer with two Intel Xeon Gold 6130 Processors (16 cores, 2.1 GHz) with 96 GB RAM. The instance dataset and the code used to perform the testing are available at the version-controlled repository https://gitlab.liu.se/eliro15/multiprocessor_scheduling_inst or via an institutional repository at https://doi.org/10.48360/etww-2281.

### 3.1 Instance conversion

To make the instance dataset accessible to a wider audience, some simplifications were made with respect to the precedence relations called dependencies in the avionics scheduling problem in [1]. Dependencies in [1] is a type of constraint that restricts the duration between the start times of a pair of tasks to be within an interval defined by a minimum and a maximum time lag. Two key differences between dependencies and the time lags used in this paper are that the order between the tasks of a dependency is not necessarily known and that the time lags can be negative. These details allow for the description of some technical constraints related to avionics scheduling. However, for the majority of the dependencies of the instances we have used, the order between the tasks of a dependency is known and the time lags are positive.

It is therefore straightforward to transform the majority of the dependencies in an instance into positive and exact time lags.

In more detail, the following transformations were made to construct a simplified problem instance. Each dependency where it is unknown in which order its two tasks are to be performed is removed. Each dependency where the minimum length is negative is removed. Each dependency where the order of its two tasks is known and its minimum length and maximum length is equal is replaced with an exact time lag. Each dependency where the order of its two tasks is known and its minimum length is positive is replaced with a positive time lag while the maximum time lag of the dependency is ignored.

## Ethics Statement

The data included in this instance dataset are primary data and do not include human subjects, animal experiments, or social media platforms.

## CRediT authorship contribution statement

**Emil Karlsson:** Conceptualization, Data curation, Formal analysis, Investigation, Methodology, Software, Validation, Visualization, Writing – original draft, Writing – review & editing. **Elina Rönnberg:** Conceptualization, Funding acquisition, Project administration, Resources, Supervision, Writing – review & editing.

## Declaration of Competing Interest

The authors declare that they have no known competing financial interests or personal relationships that could have appeared to influence the work reported in this paper.

## Data Availability

Multiprocessor scheduling instances (Original Data).
